# Effects of forage quality and particle size on feed intake and ruminoreticulum content of goats

**DOI:** 10.1093/tas/txad101

**Published:** 2023-11-20

**Authors:** Daniel Souza Lopes, Marcelo Teixeira Rodrigues, Tadeu Silva de Oliveira

**Affiliations:** Instituto Federal de Educação, Ciência e Tecnologia do Sudeste de Minas Gerais, Rio Pomba 36180-000, Brazil; Department of Animal Science, Universidade Federal de Viçosa, Viçosa 36570-000, Brazil; Laboratory of Animal Science, Universidade Estadual do Norte Fluminense, Campos dos Goytacazes 28013-602, Brazil

**Keywords:** animal behavior, fiber quality, rumen content, selectivity

## Abstract

The aim was to evaluate the effect of particle size and hay quality on feed intake, granulometric profile, and composition of the ruminoreticulum content in goats. We used 54 Alpine bucks in a completely randomized design with a factorial arrangement of 3 × 3. Treatments were a combination of Bermuda grass hay (*Cynodon dactylon*) with three quality levels: high (35 days), medium (50 days), and low (65 d) harvested at regrowth times. Were evaluated three particle sizes: small (16% ≥4.76 mm), medium (48% ≥4.76 mm), and large (75% ≥4.76 mm), which accounted for 66%, 75%, and 94% of physically effective fiber, respectively. Samples of offered diet, intake, and ruminoreticulum content were used to generate the granulometric profile. The offered diet, intake, and ruminoreticulum content presented different granulometric profiles regarding hay quality and particle size. Dry matter intake (DMI) and neutral detergent fiber intake (NDFI) increased (*P* < 0.05) when low-quality hay and large particles were offered. However, when particle size in low-quality hay was reduced, DMI and NDF decreased (*P* < 0.05). When analyzing the ruminoreticulum content (DM, NDF, peNDF, and indigestible DM), we did not observe any effect (*P* > 0.05) of hay quality or particle size on the variables. Thus, reducing hay quality and increasing particle size increase dry matter and fiber intake, presenting an interaction between forage quality and particle size. Forage quality and particle size promote intense selective behavior and chewing, which leads to a homogeneous content of particle profile in ruminoreticulum and a uniform average retention time.

## Introduction

Goats usually select their feed carefully and are considered agile eaters. They differ in feeding behavior, level of intake, diet selection, taste discrimination, and rate of eating from sheep and cattle. Because of these differences, the knowledge obtained from other ruminant species may not be able to extrapolate to goats (Lu et al., 2008; Dias-Silva and Abdalla Filho, 2021). Goats are picky, tending to select the most nutritious portions of the available feed (Van Soest, 1994). In tropical regions, grasses present high production of forage mass, but as the plant ages, cell walls become thicker and encrusted with lignin. These changes decrease forage quality and constraint microbial digestion and fermentation (Wilson, 1994; Wilson and Mertens, 1995). Thus, the reduced rates of fiber degradation in the rumen are a limitation for intake and nutrient efficiency, negatively affecting goats’ performance (Oliveira et al., 2014; Firkins, 2021). Searching for options to optimize nutrient intake is essential, but minimum levels of fiber are necessary to stimulate rumination and maintain ruminal homeostasis (Jiang et al., 2017). Fiber requirements for goats by current feeding systems have not yet been defined. However, lactating goats, in particular, require fiber to maintain a normal milk fat content and maintain the stability of the rumen ecosystem (Lu et al., 2008).

Therefore, a big challenge to the current feeding systems of small ruminants is to balance energy-dense diets with adequate amounts of dietary physically effective fiber, which is needed to prevent ruminal disorders (Zebeli et al., 2012). The optimal balance between physically effective fiber and readily degradable carbohydrates is hard to achieve. However, it is crucial to maintain proper rumen metabolism, stable metabolic health status, and enhance the system’s productivity (Allen, 1997; Zebeli et al., 2012). Assessments of dietary physical effectiveness and fiber adequacy in goats has proven to be difficult mainly due to the insufficient consideration of a feedstuff’s physical characteristics, such as particle size (Li et al., 2014). For Mertens (1997) the concept of physically effective fiber integrates information on forage quality (i.e., neutral detergent fiber contents and lignin, etc.) and structural features (i.e., particle size) that act jointly and interdependently to stabilize ruminal fermentation and acid-base balance (Allen, 1997; Humer et al., 2017). The use of this information in diet formulation thus provides a potential tool to evaluate dietary fiber adequacy in goats.

Thus, we hypothesized that there is an interaction between the chemical and physical characteristics of forage on the ingestive behavior of goats. The literature presents evidence of individual acts of these factors, but there is a scarcity of information about the interaction effects. So, the aim was to evaluate the effect of particle size and hay quality on feed intake, granulometric profile, and composition of the ruminoreticulum content in goats.

## Materials and Methods

### Animals and Diets

This study was approved by the Ethics Committee on Animal Use from the Animal Science Department of the Universidade Federal de Viçosa, protocol 61/2013. The experiment was conducted at the experimental station of the UFV, located in the municipality of Viçosa, Minas Gerais State, Brazil (20°46ʹ19″S and 2°51ʹ12″W; elev. 707 m). Climate is Cwa (tropical, high altitude), with rainy summers and dry winters according to the Köppen classification, and the average temperature is 18.5 °C, ranging between 8.2 and 28.5 °C. The average annual rainfall is 1203 mm, and 80% humidity.

We used 54 Alpine goats at 191 ± 4 days old, with an initial body mass of 24.68 ± 3.17 kg. The animals were housed in 1.0 × 1.5 m individual pens with wood slat floor and water ad libitum. The bucks were fed twice daily (08:00 am and 04:00 pm), and the meals were adjusted twice a week to keep 10% orts.

Diets comprised Bermuda grass hay (*Cynodon dactylon*) and concentrate feed (80:20, forage-to-concentrate ratio). A completely randomized design was used, in a 3 × 3 factorial arrangement, with the combination of forage harvested at three regrowth times (35 days [high-quality], 50 days [medium-quality] and 65 days [low-quality]), and three particle sizes. Large (75% ≥4.76 mm), medium (48% ≥4.76 mm), and small (16% ≥4.76 mm) particles accounted for 95, 75, and 66% of physically effective fiber, respectively. Particle size > 4 mm, which is the particle size considered peNDF (Bezerra et al. (2004)Heinriches, 2014). Large particles of hay were obtained by chopping twice in a knives and countersinks system (EN 6600, Nogueira, Brazil). Medium particles were obtained by a hay disintegrator (GTM-2001 CB, Garthen, Brazil) without sieves. Small particles were obtained by chopping in a knives and countersinks system (EN 6600, Nogueira), followed by a hammer mill (GTM-2001 CB, Garthen) fitted with a 1-cm sieve.

### Granulometric Profile

The granulometric profile of offered diet and ruminoreticulum content (wet) was determined using a mechanical stirrer model (Model BT-001, Brazil) with sieves of 4.76, 2.38, 1.19, 0.70, and 0.279 mm at maximum intensity for 1 min, and then the material retained in each sieve was weighed. The sifting process involved putting ~200 g of sample in the upper sieve. The mass in each sieve was expressed as a percentage of the sample’s total mass. We computed the particle size percentages of the fiber retained ≥ 1.19 mm as the sum of fiber retained in the sieves 4.76, 2.38, and 1.19 mm. These fractions were called physically effective fiber (≥1.19 mm), according to Bezerra et al. (2004).

### Slaughter Procedures

The experimental period lasted 37 d, with 15 d for adaptation and 22 d for samplings. Daily intake was the difference between offered feed and orts. Diet and orts samples were collected during the experimental period. The samples remained frozen at −18 °C until further analyses. At the end of the experimental period, the animals were slaughtered in order to sample ruminoreticulum content.

The slaughter was carried out at different times to represent the variation of the total ruminal mass throughout the day. One group was slaughtered 2 ho after the morning meal and the other 2 h after the afternoon meal.

The animals were stunned with a captive bolt (Blitz-Kerner, 9 × 17 mm caliber), hung upside down, and bled by sectioning the jugular and carotid arteries. After slaughter, the gastrointestinal tract of each animal was collected, except the proximal and distal portions of the esophagus, rumen, reticulum, omasum, abomasum, small intestine, and large intestine. The compartments were completely removed for weighing the content in the ruminoreticulum and the other parts of the digestive tract. The ruminoreticulum content of each animal was removed, homogenized, and sampled. Samples were divided into two aliquots. The first aliquot was ground (1 mm) for fiber analysis, and the second aliquot was used to determine the granulometric profile. Liquids, solutes, and small particles were removed by washing them under running water in a 0.977-mm sieve. Then, the material was partially dried in an oven at 55 °C for 72 h and disaggregated with a brush. The renewal rate of dry matter (DM) and neutral detergent fiber (NDF) in the rumen were estimated using ruminal content (DM or NDF kg) and DM or NDF intake (kg DM/h) ratio, according to Cannas et al. (2003).

### Chemical Analyses

Samples of hay, concentrate feed, orts, and ruminoreticulum content were analyzed for dry matter (DM, Method 967.03—AOAC, 1990), ash (Method 942.05—AOAC, 1990), crude protein (CP; [nitrogen × 6.25] Method 981.10—AOAC, 1990), ether extract (EE, Method 920.29—AOAC, 1990), calcium (Ca, Method 984.27—AOAC, 1990), and phosphorous (P, method 984.27—AOAC, 1990). Neutral detergent fiber (aNDFom) was determined by adding heat-stable alpha-amylase solution and expressed including residual ash, according to Van Soest et al. (1991). Acid detergent fiber (ADF) was obtained as proposed by Van Soest et al. (1991), and lignin by cellulose solubilization with sulfuric acid 72%. Chemical composition of the diet is shown in Table 1.

**Table 1.** Chemical composition of the feeds supplied during the experimental period

Variables	Hay quality			Concentrate
	High	Medium	Low	
Dry matter	812.0	790.2	809.3	867.3
Organic matter	900.2	883.8	899.0	957.9
Crude protein	134.1	116.1	101.0	42.1
Ether extract	24.6	20.6	19.6	41.3
Neutral detergent fiber	680.9	693.6	716.0	157.9
Acid detergent fiber	354.9	394.8	410.6	63.9
Lignin	40.7	50.0	59.2	8.0
Ash	99.8	116.2	101.0	42.1
Calcium	2.9	3.4	3.4	3.7
Phosphor	2.4	2.6	2.7	3.2

High (35 d), medium (50 d), low (65 d). All expressed as gram per kilogram, except dry matter expressed as as-fed.

### Statistical Analyses

The measured variables were staggered to the goats body mass in order to reduce the effect of size on their behavior, allowing comparisons regardless of animal mass or size. According to Kleiber (1975), the potential of general scaling can be described as


$$\begin{array}{l} y=\;\alpha X^{\beta }+\varepsilon \; \end{array}$$ (1)

where α is a steady-state expressed as a dependent variable per unit of mass raised to a power β.

Considering that lignin is a fraction of fiber (Van Soest, 1994) and assuming that the removal of indigestible matter from the ruminoreticulum is not confused with the loss of mass through digestion (Ellis, 1978), the renewal rate of lignin staggered to the animal mass was used to estimate the fractional passage rate. The renewal rate of lignin was directly estimated using the ratio between the lignin retained in the rumen and lignin intake (Qlig/Clig), applying a robust nonlinear regression with a standard deviation of σ = 0.5d.

The parameters of Equation (1) were estimated using a robust nonlinear regression procedure (PROC NLIN) and the least-squares method based on the Marquardt algorithm implemented in the SAS program (SAS University Edition, SAS Institute Inc., Cary, NC, USA). An initial estimate of standard deviation (σ) and an adjustment constant (4.685) were used to meet the robustness criterion as proposed by Beaton and Tukey (1974). Means were compared through the Student–Newman–Keuls (SNK) test at 0.05 probability using the PROC MIXED from SAS.

## Results

### Granulometric Profile

**Figure 1.**
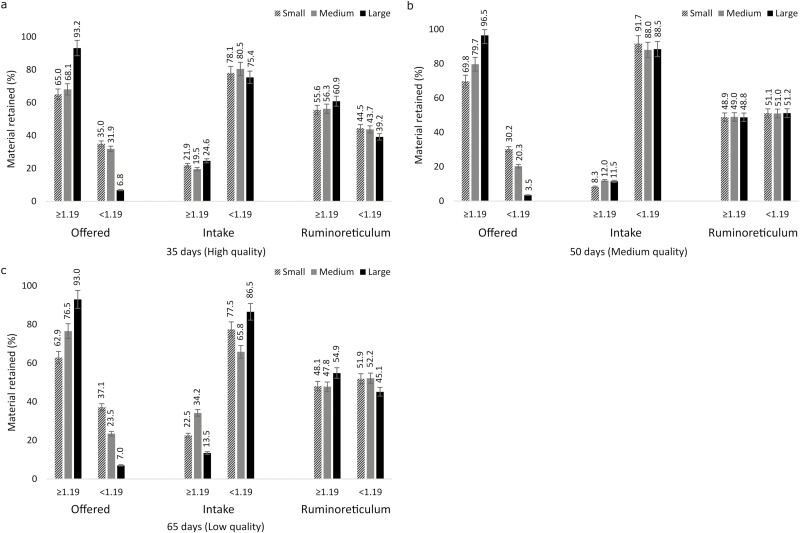
Granulometric profile of offered feed, intake (offered minus orts), and ruminoreticulum content to hay qualities (a) high, (b) medium, and (c) low with particles size ≥ 1.19 and < 1.19 mm.

The percentage of dietary particles retained in the 1.19 mm sieve was >60%, regardless of hay quality. This trend was also observed for the intake of particles < 1.19 mm (Figure 1). Regardless of particle size from the diet, the ruminoreticulum granulometric profile was similar to the forage qualities. Mean values for physically effective fiber (pef ≥ 1.19 mm) of offered diet were 65.9%, 74.8%, and 94.2% for small, medium, and large particles, respectively. Values for pef ranged between 50.8%, 51.0%, and 52.5% for small, medium, and large particles, respectively, in the ruminoreticulum. Ruminoreticulum content showed a lower proportion of particles retained in the < 1.19 mm sieve only for high-quality hay, but there was no difference for the others (Figure 1).

### Intake, Ruminoreticulum Content Composition, and Average Retention Time

There was no interaction effect between particle size and hay quality (*P* > 0.05) on the analyzed variables (Tables 3 and 4). Particle size and hay quality affected (*P* < 0.05) DM, NDF, peNDF, and lignin intake (Tables 2 and 3). The DMI increased when medium particles (*P* < 0.001) were offered. The particle size reduction in hay (small and medium) increased NDF intake (NDFI) (*P* < 0.001); however, peNDF intake decreased (*P* < 0.001). Lignin followed a similar behavior to NDFI (Tables 2 and 3). The DM, NDF, peNDF, and lignin intake increased when low-quality hay (*P* < 0.05) (Tables 2 and 3).

**Table 2.** Parameter estimates α and β related to each parameter studied

Variables	A ± S D	CI (0.95)		B ± S D	CI (0.95)	
		Lower	Upper		Lower	Upper
Intake						
DM total	0.93 ± 0.70	−0.48	2.34	1.97 ± 0.2	1.51	2.42
DM hay	0.63 ± 0.48	−0.34	1.61	1.98 ± 0.23	1.52	2.45
NDF	0.41 ± 0.25	−0.09	0.91	2.04 ± 0.18	1.67	2.4
peNDF total	3.16 ± 3.7	−4.2	10.51	1.24 ± 0.35	0.53	1.95
peNDF hay	3.23 ± 3.78	−4.4	10.83	1.23 ± 0.36	0.51	1.94
Lignin	0.03 ± 0.03	−0.03	0.1	1.97 ± 0.29	1.37	2.53
Ruminoreticulum content						
DM	1.64 ± 1.13	−0.63	3.91	1.81 ± 0.21	1.39	2.22
NDF	1.1 ± 0.8	−0.49	2.73	1.72 ± 0.22	1.28	2.16
peNDF	0.68 ± 0.59	−0.52	1.88	1.67 ± 0.27	1.14	2.2
Lignin	0.32 ± 0.26	−0.2	0.85	1.44 ± 0.24	0.95	1.93
iDM	0.76 ± 0.62	−0.49	2.0	1.45 ± 0.25	0.95	1.94
Mean retention time						
MRT_DM_	25.73 ± 0.74	24.24	27.2	−0.65 ± 0.49	−1.64	0.34
MRT_NDF_	23.75 ± 0.76	22.22	25.28	−0.36 ± 0.58	−1.52	0.79

CI = confidence interval; SD = standard deviation of the parameter; DM = dry matter; NDF = neutral detergent fiber; peNDF = physically effective neutral detergent fiber; iDM = indigestible dry matter; MRT_DM_ = mean retention time of dry matter; MRT_NDF_ = mean retention time of neutral detergent fiber. Equation: $y=\alpha X^{\beta }+\varepsilon$. The α is a steady-state expressed as a dependent variable per unit of mass raised to a power_β_.

**Table 3.** Dry matter and fiber compounds intake as a function of particle size and forage quality

Variables	Particle size			SEM	*P*-value
	Small	Medium	Large		
DMI, g kg^−1.97^.d^−1^	0.92^b^	1.04^a^	0.86^b^	0.038	<0.001
NDF, g kg^−2.04^.d^−1^	0.41^a^	0.40^a^	0.38^b^	0.006	<0.001
peNDF, g kg^−1.24^.d^−1^	2.41^b^	2.77^b^	4.17^a^	0.405	<0.001
Lignin, g kg^−1.97^.d^−1^	0.04^a^	0.04^a^	0.03^b^	0.001	<0.001

	Forage quality				
	High	Medium	Low		
DMI, g kg^−1.97^.d^−1^	0.88^b^	0.89^b^	0.95^a^	0.017	0.002
NDF, g kg^−2.04^.d^−1^	0.39^b^	0.38^b^	0.42^a^	0.009	0.002
peNDF, g kg^−1.24^.d^−1^	2.69^b^	2.69^b^	3.48^a^	0.205	0.003
Lignin, g kg^−1.97^.d^−1^	0.03^b^	0.03^b^	0.05^a^	0.004	0.002

High (35 d), medium (50 d), low (65 d), SEM = standard error of the means, FQ = forage qualit, PS = particle size, DM = dry matter, NDF = neutral detergent fiber, peNDF = Physically effective neutral detergent fiber.

Means followed by the different letters differ significantly by the Student–Neuman–Keuls test (*P* < 0.05).

**Table 4:** Ruminoreticulum content and composition, mean time of rumen renewal for dry matter and neutral detergent fiber as a function of particle size and forage quality

Variables	Particle size			SEM	*P*-value
	Small	Medium	Large		
DM, g kg^−1.81^	1.62	1.55	1.53	0.021	0.289
NDF, g kg^−1.72^	1.15	1.05	1.03	0.026	0.984
peNDF, g kg^−1.67^	0.67	0.63	0.60	0.013	0.773
iDM, g kg^−1.45^	23.23	21.79	19.34	0.812	0.888
Lignin, g kg^−1.44^	0.32^a^	0.31^b^	0.31^b^	0.001	0.025
DM renewal time (h)	25.94	25.56	26.31	0.143	0.358
NDF renewal time (h)	23.81	23.64	24.72	0.256	0.594

Forage quality					
	High	Medium	Low		
DM, g kg^−1.81^	1.56	1.56	1.58	0.004	0.526
NDF, g kg^−1.72^	1.07	1.05	1.12	0.015	0.515
peNDF, g kg^−1.67^	0.71	0.59	0.59	0.031	0.199
iDM, g kg^−1.45^	21.97	19.74	22.65	0.661	0.252
Lignin, g kg^−1.44^	0.28^b^	0.32^a^	0.34^a^	0.012	0.024
DM renewal time (h)	25.83	27.68	24.29	0.671	0.754
NDF renewal time (h)	23.95	25.28	22.95	0.470	0.864

High (35 d), medium (50 d), low (65), SEM = standard error of the means, FQ = forage quality, PS = particle size, DM = dry matter, NDF = neutral detergent fiber, peNDF = physically effective neutral detergent fiber, iDM = indigestible dry matter.

Means followed by the different letters differ significantly by the Student–Neuman–Keuls test (*P* < 0.05).

When analyzing the ruminoreticulum content (DM, NDF, peNDF, and iDM), we did not observe any effect (*P* > 0.05) of hay quality or particle size on these variables (Tables 2 and 4). However, lignin content in ruminoreticulum was low for high-quality hay (*P* = 0.024) and medium and large particles (*P* = 0.025) (Tables 2 and 4). Forage quality and particle size did not influence (*P* > 0.05) the renewal rate of DM and NDF in the goats ruminoreticulum (Tables 2 and 4).

## Discussion

### Granulometric Profile

The 1.18 mm sieve is widely used as the size in which feed particles are retained in the rumen, considered physically effective for ruminants (Mertens, 1997; Beauchemin et al., 2003; Maulfair et al., 2011). For Yang et al. (2001) and Maulfair et al. (2011), the retention of particles in < 1.19 mm sieves is considered a critical point for possible escape from the ruminoreticulum compartment. In our study, more than 60% of the offered hay (regardless of quality) was retained in ≥ 1.19 mm sieves (Figure 1). When analyzing the granulometric profile of high-quality hay, we observed that 93.2% of large particles were retained in the ≥ 1.19 mm sieves, medium particles 68.1%, and small particles 65%. However, more than 70% of hay intake was < 1.19 mm particles, which increased the escape of < 1.19 mm particles (Figure 1). The selective feeding behavior of goats explains this fact. The medium and low-quality hays followed the same pattern, except for the intake of < 1.19 mm particles. The intake of these particles was higher for the medium-quality hay, probably because the initial mass of the hay reduced its size due to the processing of the material.

Different granulometric profiles were found for different particle sizes, and goats easily selected large particles. According to Morand-Fehr (2003) and Findeisen et al. (2021), goats prefer large particles to small particles because the larger size is easier to bite. However, this fact was not observed for all conditions in the present study. The preference for large particles was observed when we offered mainly small particles, and small particle preference was detected when we offered medium and large particles (Figure 1). According to Van Soest (1994), feeding selectivity is a strategy of goats to seek the most nutritious parts of plants and increase the passage rate to the less nutritious parts.

Ruminants clearly prefer feeds of better nutritional quality (high concentration of nutrients). However, according to Baumont et al. (2000), in conditions where a diet with high energy concentration is available (feedlot), animals do not always select only the fractions rich in readily available energy, they also select fibrous parts to maintain a stable rumen function. The granulometric profiles of the intake of medium and low-quality hays presented more large particles than high-quality hay (Figure 1). This is probably a response to the high lignification of older plants (Van Soest, 1994), which implies more resistance to being sheared. Regardless of particle size, feed intake, and particle profiles of ruminoreticulum content for high, medium, and low-quality hays (Figure 1) reinforce the goat’s selective feeding behavior.

A high proportion of ≥ 1.19 mm particles were retained in the ruminoreticulum for the high-quality hay (Figure 1). This may be due to the high resistance to digestion flow (Poppi et al., 1980). In addition, a minimal content of physically effective fiber (pef > 1.19 mm) is necessary to maintain ruminal function (Mertens, 1997; Zebeli et al., 2012). There was no difference in percentages of particles retained in ≥ 1.19 mm and < 1.19 mm sieves for medium and low-quality hays (Figure 1).

### Intake, ruminoreticulum content composition, and average retention time

A decrease in feed intake was expected as the plant aged. Consequently, the digestibility decreases, providing gastric filler (Moyo et al., 2019). However, we observed in our study a higher DM and peNDF intake for low-quality hay (65 days) with medium particles than medium (50 days) and high-quality (30 days) hays. This fact suggests that as grass ages, the intake increases to meet the nutrient requirements, as long as hay is not excessively chopped. It is probably because such processing hinders the selection, obliging them to ingest lignified tissues and possibly increasing the passage rate (Van Soest, 1994). In our study, the animals consumed more lignin when fed low-quality hay and small and medium particles (Table 3). It is possible that bucks spent more time chewing because of the large particle size and other characteristics of the physiologically aged material. This activity contributed to a rapid reduction of the retained particles, promoting the escape from ruminoreticulum (Hummel et al., 2018).

The fiber fraction of ruminoreticulum content did not differ among treatments, except for lignin. A possible explanation is the selective behavior and chewing activity, including rumination, which caused the intake of small particles regardless of the offered diet (Figure 1). Jalali et al. (2012) compared the intake and ingestive behavior of goats, sheep, and llamas. The authors pointed out that goats spent 36.19% more time eating, 31.4% more time chewing, and 28.55% more time ruminating per kilogram of ingested DM than sheep submitted to the same diets. Goats have a more intense selective feeding behavior, chewing activity, and rumination than other ruminants. These behaviors reduce particles after and before swallowing, generating an almost uniform ruminal particle concentration. Therefore, the fibrous material in the ruminoreticulum is less heterogeneous in terms of particle sizes that are eligible for escaping the ruminal environment compared the intake and ingestive behavior of goats, sheep, and llamas. The authors pointed out that goats spent 36.19% more time eating, 31.4% more time chewing, and 28.55% more time ruminating per kilogram of ingested DM than sheep submitted to the same diets. Goats have a more intense selective feeding behavior, chewing activity, and rumination than other ruminants. These behaviors reduce particles after and before swallowing, generating an almost uniform ruminal particle concentration. Therefore, the fibrous material in the ruminoreticulum is less heterogeneous in terms of particle sizes that are eligible for escaping the ruminal environment(Moyo et al., 2019). Lignin in the ruminoreticulum content was different for animals that received hay with large particle size, increasing lignin accumulation with low-quality forage (Table 4). This was expected since, in these cases, there is also an increase in DMI, even with low-quality feeds.

In theory, low-quality forage has high lignin concentration in the fibrous fraction and high rumen retention time for DM and NDF (Baumont et al., 2000), which would lead to contrary results to those observed in our study. A possible explanation for our results may be related to the goat’s perception of particles with high shear resistance, stimulating more intense chewing on these particles, and leading to a rapid size reduction of the material.

In conclusion, reducing hay quality and increasing particle size increase dry matter and fiber intake, presenting an interaction between forage quality and particle size. Forage quality and particle size lead to intense selective and chewing behavior, which leads to a homogeneous content of particle profile in ruminoreticulum and a uniform average retention time.
